# Biased and Unbiased Methods for the Detection of Off-Target Cleavage by CRISPR/Cas9: An Overview

**DOI:** 10.3390/ijms17091507

**Published:** 2016-09-08

**Authors:** Francisco Martin, Sabina Sánchez-Hernández, Alejandra Gutiérrez-Guerrero, Javier Pinedo-Gomez, Karim Benabdellah

**Affiliations:** 1 Genomic Medicine Department, GENYO—Centre for Genomics and Oncological Research Pfizer-Universidad de Granada-Junta de Andalucía, Avda de la Ilustración 114, 18007 Granada, Spain; sabina.sanchez@genyo.es (S.S.-H.); alejandra.gutierrez@genyo.es (A.-G.G.); javier.pinedo@genyo.es (J.P.-G.); 2 LentiStem Biotech, GENYO, Avda de la Ilustración 114, 18007 Granada, Spain

**Keywords:** specific nucleases, CRISPR/cas9, off-target sites, real target cells, unbiased, biased, CHIPSeq, IDLVs, GUIDE-seq, LAM-HTGTS

## Abstract

The clustered regularly interspaced short palindromic repeat (CRISPR)-associated protein 9 endonuclease (Cas9) derived from bacterial adaptive immune systems is a revolutionary tool used in both basic and applied science. It is a versatile system that enables the genome of different species to be modified by generating double strand breaks (DSBs) at specific locations. However, all of the CRISPR/Cas9 systems can also produce DSBs at off-target sites that differ substantially from on-target sites. The generation of DSBs in locations outside the intended site can produce mutations that need to be carefully monitored, especially when using these tools for therapeutic purposes. However, off-target analyses of the CRISPR/Cas9 system have been very challenging, particularly when performed directly in cells. In this manuscript, we review the different strategies developed to identify off-targets generated by CRISPR/cas9 systems and other specific nucleases (ZFNs, TALENs) in real target cells.

## 1. Introduction: The CRISPR/CAS9 Genome Editing System and the Off-Targets

The CRISPR/Cas9 system derived from bacterial adaptive immune systems [1,2,3] is one of the most powerful genome editing technology [4,5]. Unlike other specific nucleases (SNs) that rely onprotein-DNA recognition (ZFNs, TALEN and Meganucleases), the specificity of the CRISPR/Cas9 system depends on RNA-DNA recognition. The guide RNA (gRNA) hybridizes with a ~20 nucleotide sequence next to a protospacer-adjacent motif (PAM) and enables the CAS9 endonuclease to cleavage the target site. Changing the nucleotide composition of the 5′ end of the gRNA modifies the site targeted by the Cas9 endonuclease. Different Cas9 nucleases derived from *Streptococcus pyogenes*, *Neisseria meningitidis*, *Staphylococcus aerous*, *Streptococcus therophilus*, and *Treponema denticola* have been used to develop efficient genome editing tools. Each CRISPR/Cas9 system differs in terms of gRNA design and PAM requirements in order to achieve specific DSBs. However, all of the CRISPR/Cas9 systems can also generate DSBs mutation at off-target sites that differ by up to several nucleotides from in-target sites. The generation of DSBs in off-target genomic locations can produce insertions and deletions (indels) as well as translocations that need to be carefully monitored [6]. The understanding and prevention of the off-target effects are fundamental for research and therapeutic purposes.

The generations of DSBs in off-target locations can occur because the Cas9 protein bind to a PAM-like sequence and/or the gRNA bind to sequences that are similar to the target site. However, finding off-targets of the CRISPR/Cas9 system and other specific nucleases have been very challenging. In this review, we focus on the different strategies developed to identify the off-targets generated “in vivo”, in real target cells by CRISPR/cas9 systems, and other specific nucleases (ZFNs, TALENs). “In vitro” off-target studies can be revised elsewhere [7,8].

## 2. Biased Detection of Off-Target Cleavage by CRISPR/Cas9

**Figure 1:**
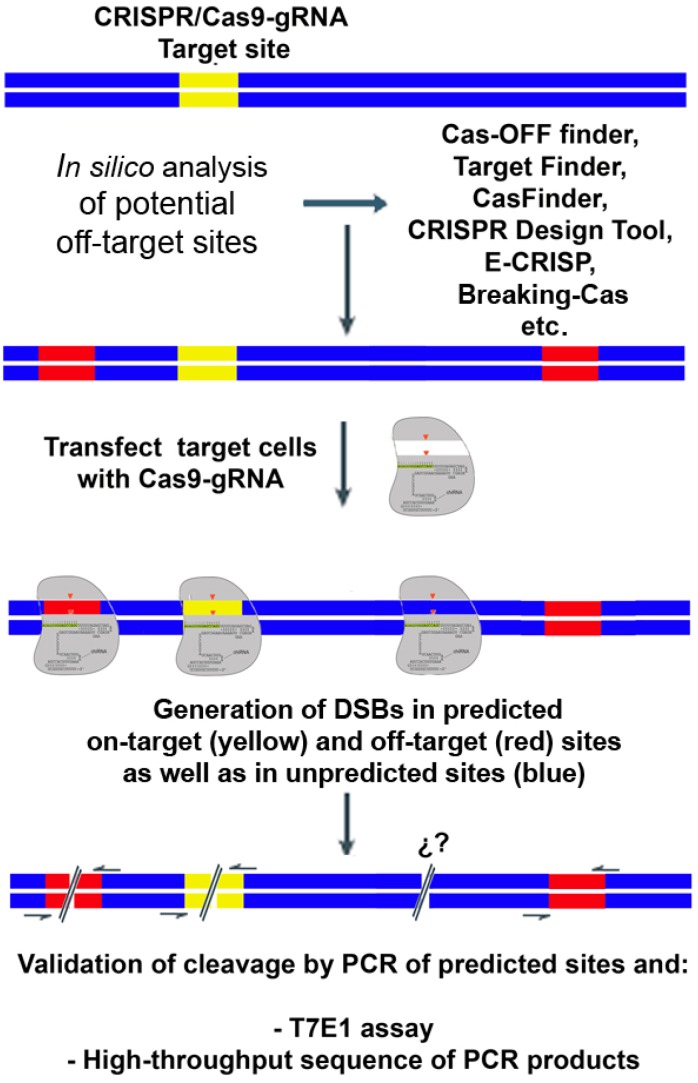
Biased off-target analysis using ChipSeq. Potential off-target sites (red) are predicted in silico by different programs freely available on the internet. The cells are transfected with the Cas9 and the gRNA, which generates DSBs at the in-target site (yellow) and in the off-target sites (red and blue). In-target and predicted off-target sites (red) can be analyzed by PCR and T7E1 assays or by high-throughput sequencing. However, the DSBs generated outside the predicted sites (blue) are undetectable by these methods.

The first strategy used to identify off-target sites of the CRISPR/Cas9 systems focused on identifying potential binding sites of the gRNA by in silico prediction. The predicted off-targets are then analyzed by standards methods: PCR of the predicted off-target site and analysis of heteroduplex formation or high-throughput sequencing of the PCR products (Figure 1). Fu et al. [9] published the first study to point out that the CRISPR/Cas9 system can generate high levels of off-target sites under certain conditions. Since then, various research groups have produced abundant data on off-target sites from different systems using different strategies [10,11,12,13,14,15,16,17]. For example, Cho et al. [11] used a bioinformatic approach to search for potential binding sites for gRNAs targeting the *CCR5* gene, which is a target for the treatment of AIDS. The potential off-target sites were then empirically verified by PCR and T7E1assays. They tested four sites in the human genome, each of which carried 3-base mismatches, as compared with the on-target site. Although the T7EI assays showed no off-target sites with this system (assay sensitivity, ~0.5%), some were found using more sensitive detection methods [12].

The data generated by different research groups on the off-target cleavage of the CRISPR/cas9 systems were used for the generation of more accurate algorithms to detect such sites. Systems such as the Cas-OF Finder (available online: http://www.rgenome.net/cas-offinder/) [18], the Feng Zhang lab’s Target Finder (available online: http://crispr.mit.edu/), the CasFinder (available online: http://arep.med.harvard.edu/CasFinder/), the CRISPR Design Tool (http://www.genome-engineering.org/) [13], E-CRISP (available online: http://www.e-crisp.org/E-CRISP/) [19], and the Breaking-cas (available online: http://bioinfogp.cnb.csic.es/tools/breakingcas/) [20]. These algorithms have been used by several research groups to define the potential off-target sites. For example, Chen et al. targeted different genes in human pluripotent stem cell (hPSC) lines. The authors used the Feng Zhang lab’s Target Finder software (available online: http://crispr.mit.edu) and analyze a total of 114 potential off-target sites, none of which showed any indel formation [16]. Other authors have come up with different results using deep sequencing assays to identify a limited number of off-target cleavages [9,13,17,21] and have generally concluded that high-frequency mutagenesis is possible even at locations quite different from the intended target site. For example, although the optimal PAM sequence recognized by Cas9 derived from *S. pyogenes* is 5′-NGG-3′, it can recognize and cleave sites with a 5′-NAG-3′or 5′-NGA-3′ sequence which can, though less efficiently, act as PAM-like motifs. On the other hand, up to 6 nucleotides mismatches and 1 bp bulge indel can be tolerated between the gRNA and the targeted sequence. Their data also show that mismatches are better tolerated at 5′ end of the 20-nt targeting region than at the 3′ end.

In summary, these studies revealed a highly complex picture of Cas9 specificity. The effects of a single mismatch are not always predictable on the basis of only their position in the gRNA. Additionally, the genomic or epigenomic context, or both, might also affect the cleavage frequency. These factors make it very difficult to develop an algorithm capable of identifying all potential off-target sites. Therefore, it can be concluded that the use of biased methods should be accompanied with other technologies that allow unbiased detection of off-target sites.

## 3. Unbiased Detection of Off-Target Cleavage by CRISPR/Cas9 in Real Target Cells

**Figure 2:**
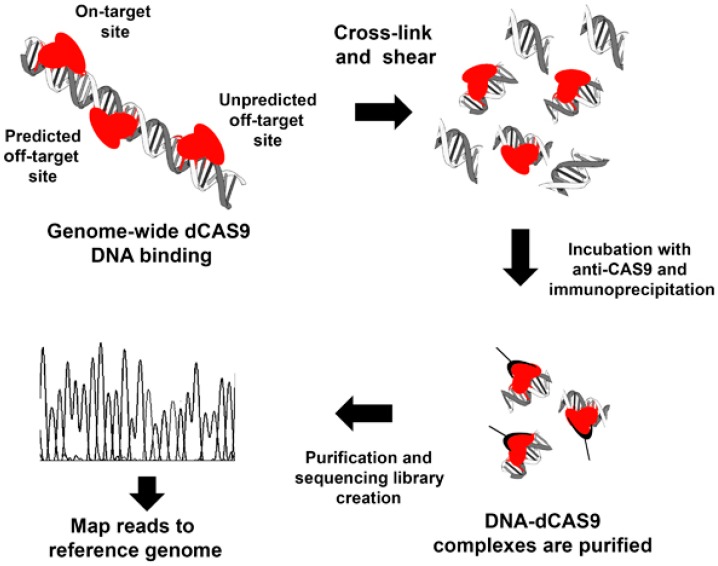
Off-target analysis by ChipSeq using dCas9. Endonuclease-defective Cas9 (dCas9) are transfected into the target cells. dCas9 remains stably bound to target sites due to the lack of endonuclease activity. Target cell DNA is extracted and sheared to obtain small DNA fragments. Some of these DNA fragments have bound dCas9 and can be purified using monoclonal Cas9 antibodies. The DNA fragments are further purified to construct a library of DNA sequences that bind to that particular Cas9-gRNA combination in the cell analyzed.

**Figure 3:**
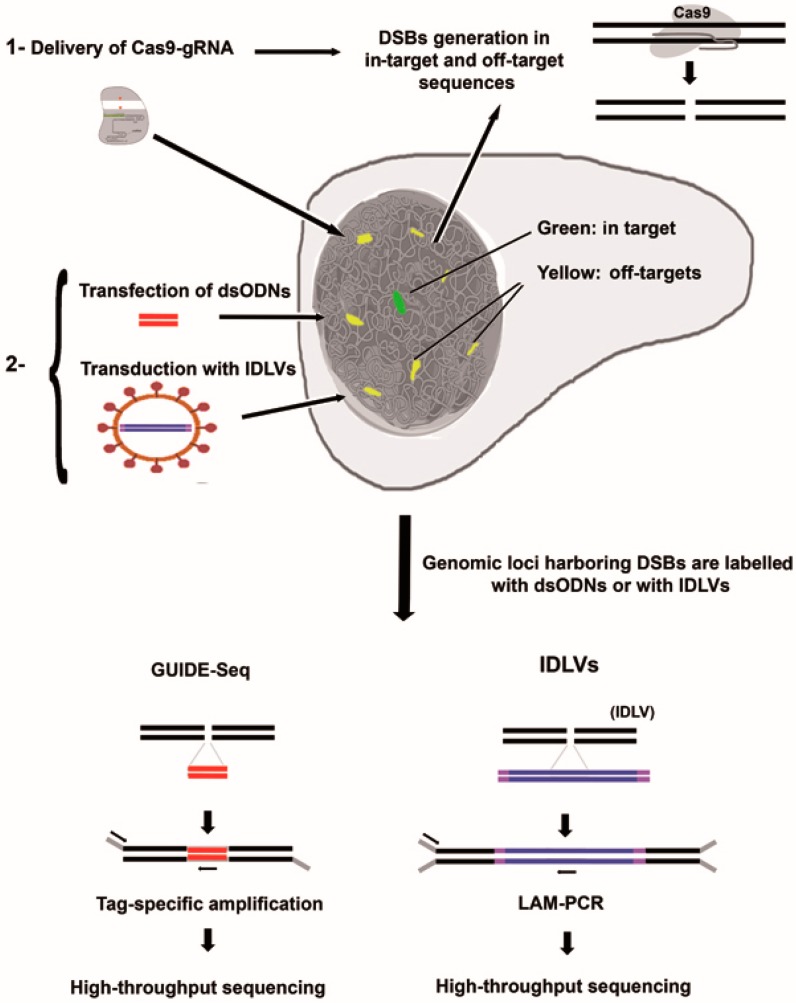
Unbiased methods for detection of in vivo DSBs generated by the CRSIPR/Cas9 system. Both methods consist in a tandem procedure: (1) The Cas9 and the gRNA are delivered into the target cells to induce the DSBs (top). These DSBs are generated at the in-target site (green) and at the off-target sites (yellow); (2) After 24–48 h, depending on the system used to deliver Cas9-gRNA complexes, the dsODNs (GUIDE-seq) or the IDLVs particles (IDLV-capture) are delivered. The dsODNs and the IDLVs integrate into the DSBs and label the genomic locations where the Cas9-gRNA cleaves in an unbiased manner and in real cell targets. The genomic locations are identified by tag-specific amplification (GUIDE-seq) or LAM-PCR (IDLV-capture) and high-throughput sequencing.

Unbiased off-target analysis needs to detect unintended cleavage sites on live cells in a totally unrestricted way (Figure 2 and Figure 3). Thus, we need to be able to directly detect mutations generated in the target cells due to DSBs produced by the CRISPR/Cas9-gRNA system outside their target locus. Below, we describe the new methodologies that are uncovering the real off-target effects of the CRISPR/Cas9 system and other genome editing technologies.

### 3.1. Cross-Linking Chromatin Immunoprecipitation of Endonuclease-Mutant Cas9 (ChIP-dCas9)

The idea behind this strategy is to detect all the binding sites of a certain gRNA-Cas9 pair. This indirect method assumes that the presence of Cas9 on a certain locus will always generate DSBs. Cross-linking chromatin immunoprecipitation (ChIP) detects the in situ interaction of proteins with DNA [22,23]. The combination of ChIP with second-generation DNA-sequencing technology (ChIP-seq) enables the detection of sites where certain proteins are bound to the DNA at the genome levels. The methodology involves the use of a catalytic inactive version of the Cas9 (dCas9) to ensure stable binding of the gRNA-Cas9 complex to the target locus. ChIP-seq of cells transfected with the dCas9-gRNA system produces a clear readout of Cas9 binding sites in the genome (Figure 2), which include both in-target and off-target sites. However, while some studies have confirmed cleavage at dCas9 binding sites, others found little or no genome editing in the Cas9-bound-DNA sites. For example, Cenci et al. found that, of the 43 ChIP-seq predicted sites for a Cas9-gRNA targeting the *Trp53* locus, only the in-target site and one off-target were confirmed [24]. In another study, Kuscu et al. [25] mapped genome-wide binding sites of catalytically inactive Cas9 (dCas9) with 12 different single guide RNAs (sgRNAs) and found significant cleavage at almost 50% of the predicted off-target sites. Therefore, although this method could give important information on potential CRISPR/Cas9 system binding sites, it does not always correlate with off-target sites. Some research groups have pointed to the use of the mutated Cas9 (dCas9) rather than native Cas9 proteins as a major limitation which could alter specificity [26]. Therefore, as can be seen below, most efforts to detect the CRISPR/Cas9 system off-target sites have focused on detecting DSBs generated by the Cas9-gRNAs. Thus, we next discuss IDLV capture, GUIDE-seq, and LAM-HTGTS technologies. All methods are able to detect genome-wide DSBs generated by specific nucleases in an unbiased manner and in real cellular targets.

### 3.2. Integrative-Deficient Lentiviral Vectors (IDLV) Capture

IDLV capture was the first genome-wide unbiased strategy to measure the off-target frequency of zinc finger nucleases (ZFNs) [27]. IDLV, as their integrase-competent LVs counterparts, can enter in most target cells in a highly efficient way. However, as IDLVs are integrase-deficient, they remain in the nuclei of target cells as episomal DNAs. Interestingly, episomal IDLVs can be integrated into DSBs sites and were used by Gabriel et al. [27] as a tool to develop the first unbiased approach to detecting off-target sites generated by ZFNs (Figure 3, right). This approach has also been used to detect off-target sites of other specific nucleases, including the CRISPR/Cas9 system [28,29]. The main advantage of this strategy lies in the highly efficient way in which IDLVs enter into the nucleus of important target cells, including difficult-to-transfect primary human cells. However, because a large proportion of IDLV insertions are found in non-relevant genomic sites, it is important to perform the experiments with the appropriate controls.

In an elegant study, Wang et al. [29] compared the efficiency and specificity of CRISPR/Cas9 and TALENs targeting the same genomic regions in the *WAS* and *TAT* genes. Unlike cells transduced with IDLVs alone, cells transduced with endonucleases and IDLVs showed clustered IDLV integration sites (CLISs) within 60 bp from the cleavage site. In fact, several CLISs were identified for both *WAS* and *TAT* locus, with seven of the nine off-target sites identified being confirmed by the Surveyor assay. This method is able to detect off-target cleavage sites with a frequency of 1%. Interestingly, Wang et al. [29] found off-target sites harboring one-base bulge or up to 13 mismatches between the gRNA and its genomic target. Similar studies were performed by Osborn et al. [26] that targeted the *TRAC* locus using CRISPR/cas9-, TALEN-, and MegaTAL-specific nucleases. In contrast to the work carried out by Wang et al. [29], in this study, Osborn et al. only found IDLVs CLISs in the *TRAC* locus using the CRISPR/Cas9 and TALEN systems and were unable to detect off-target cleavage activity. Only the MegaTAL nuclease showed CLISs in several off-target sites in addition to the *TRAC* locus. This may indicate a lower sensitivity of the Osborn et al. experiments, a superior design of the CRISPR/Cas9 and TALEN systems, or both.

### 3.3. Detection of Off-Target Cleavage by CRISRP-Cas9 Using GUIDE-seq Methods

Keith Joung’s team recently developed a revolutionary new platform called GUIDE-seq (genome-wide unbiased identification of DSBs evaluated by sequencing) [30]. The method requires transfecting edited cells with small, modified double-stranded oligonucleotides (dsODNs) that are incorporated into existing DSBs in a cell’s DNA (Figure 3, left). The dsODNs is used as a tag to amplify the genomic areas harboring DSBs that are later identified by high-throughput sequencing. DSBs are identified by mapping tag-amplified reads with a reference genome. GUIDE-seq is highly sensitive, as it is capable of detecting off-target sites that occur at a frequency of 0.1% in a cell population. This method was firstly used to detect the off-target sites of 10 different gRNAs targeted to various human genes in two cell types. Keith Joung’s team found off-targets that harbored up to six mismatches between the putative gRNA binding sites and the real target. They also detected non-canonical PAMs, including previously described NGA, NAA, NGT, NGC, and NCG sequences. Like previous off-target studies that have used IDLVs, they concluded that the CRISPR/Cas9 system can generate numerous off-target cleavages that were not predicted by in silico analysis. In fact, their analyses show that some off-target sites are even more frequent than the DSBs found at on-target sites. The principal advantage of the GUIDE-seq technology is the precision with which dsODNs capture can define off-target sites and the direct correlation between the number of reads at a given site and the frequency of DSBs generated by the CRISPR/Cas9-gRNA system.

When the data generated by GUIDE-seq were compared to those produced by ChIP-seq using the same CRISPR/Cas9 systems, the authors found very little overlaps [30]. They concluded that very few dCas9 off-target biding site described by ChIP-seq are bona fide Cas9 off-target cleavage sites [30]. Several manuscripts have used the GUIGE-Seq to investigate real off-target sites of the CRISPR/Cas9 systems [31,32,33]. The main limitation of the GUIDE-seq technology is that it requires a high level of transfection efficiency on the target cells, which limit the use of this method in some cell types.

### 3.4. LAM-PCR-Based High-Throughput Genome-Wide Translocation Sequencing (LAM-HTGTS)

LAM-HTGTS [34,35] is an unbiased-genome-wide-based method for the detection of off-target which relies on the identification of chromosomal translocation that may arise by fusion of ends of two DNA DSBs generated by SNs. The strategy consists inthe generation of libraries containing genome-wide “bait-prey” junctions that represent the junctions of chromosomal translocations. The bait-prey junctions are cloned from genomic DNA using LAM-PCR, ligated to adapters of known sequence, perform a PCR to amplify the library and sequenced. A bioinformatic pipeline identifies the sequences (preys) corresponding to the chromosome points that lead to translocations due to DSBs generation by the SNs.

Unlike the other unbiased methods described, LAM-HTGTS not only detects potential off-target sites but also reveal collateral damage as a consequence of these unspecific cleavages (recurrent translocations). The major limitation of this approach is the scarcity of the translocation events and the high dependency of the 3D genome organization since chromosomal translocations occur preferentially in chromosomes that are in close proximity.

### 3.5. Whole Genome Sequencing (WGS)

In theory, as the entire genome of a cell can now be sequenced, the WGS of edited cells could be used to study CRISPR/Cas9 specificity [36,37]. Indeed, by sequencing the whole genome before and after genome editing, off-target sites can be determined by a simple analysis of the new mutations that have been generated outside the intended locus, as compared with the original population. This method is useful for the analysis of single cells, clones, and F1 genome-edited animals. However, WGS, which only detects higher frequency off-target sites, lacks the sensibility required to detect off-target sites in bulk populations [36,38].

## 4. Conclusions

The precise identification of CRISPR/Cas9 cleavage sites in real cell targets, including in-target and off-target sites, is of crucial importance in order to understand the potential side effects of genome editing technologies and, at the same time, to design new systems with enhanced specificity. Two complementary strategies have been used in order to detect potential off-target sites: (1) biased methods that rely on bioinformatics algorithms to predict potential off-target sites and (2) unbiased methods that identify genome-wide DSBs generated by the SNs. Biased methods are, at the moment, unable to detect a large number of off-target sites and have become therefore a method to complement unbiased methods. Indeed, several unbiased technologies have been described (WGS, IDLV capture, GUIDE-seq, and LAM-HTGTS) that have shed light to the real specificity of several SNs, confirming some off-target sites predicted by bioinformatic algorithms and uncovering many more. The results obtained by these unbiased methods are helping to understand the mechanism by which CRISPR/Cas9 cleavage their target site and have become of crucial importance for the development of improved systems.
